# Potential applications of peptide nucleic acid in biomedical domain

**DOI:** 10.1002/eng2.12238

**Published:** 2020-07-24

**Authors:** Kshitij RB Singh, Parikipandla Sridevi, Ravindra Pratap Singh

**Affiliations:** ^1^ Department of Biotechnology, Faculty of Science Indira Gandhi National Tribal University Amarkantak Madhya Pradesh 484887 India

**Keywords:** Biomedical application, Biosensors, Diagnosis, PNA properties, PNA, Therapeutics

## Abstract

Peptide Nucleic Acid (PNA) are DNA/RNA synthetic analogs with 2‐([2‐aminoethyl] amino) acetic acid backbone. They partake unique antisense and antigene properties, just due to its inhibitory effect on transcription and translation; they also undergo complementary binding to RNA/DNA with high affinity and specificity. Hence, to date, many methods utilizing PNA for diagnosis and treatment of various diseases namely cancer, AIDS, human papillomavirus, and so on, have been designed and developed. They are being used widely in polymerase chain reaction modulation/mutation, fluorescent in‐situ hybridization, and in microarray as a probe; they are also utilized in many in‐vitro and in‐vivo assays and for developing micro and nano‐sized biosensor/chip/array technologies. Earlier reviews, focused only on PNA properties, structure, and modifications related to diagnostics and therapeutics; our review emphasizes on PNA properties and synthesis along with its potential applications in diagnosis and therapeutics. Furthermore, prospects in biomedical applications of PNAs are being discussed in depth.

## Graphical abstract

Peptide nucleic acids are DNA/RNA synthetic analogs. They show unique stability and hybridization properties and have enormous biomedical applications for diagnostics (utilizing biosensors, microarray, and so on) and therapeutics. Hence, this review emphasizes the PNAs biomedical application along with its properties, synthesis, and prospects.

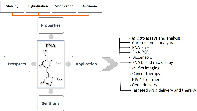

## 1 INTRODUCTION

**FIGURE 1:**
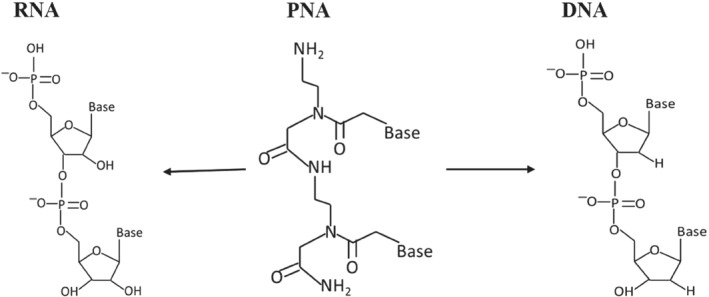
Structural comparison of PNA with RNA and DNA

Peptide nucleic acid (PNA) is a synthetic analog of DNA/RNA (Figure 1) with 2‐([2‐Aminoethyl] amino) acetic acid backbone, which results in achiral and uncharged mimic. It is regarded as DNA but has a neutral peptide backbone on the place of a negatively charged sugar‐phosphate backbone and due to their unique physical and chemical properties such as resistant to enzymatic degradation inside living cells have offered them with extensive consideration in various fields for application.^1^, ^2^, ^3^ It has shown promising applications as a biomolecular tool, antisense and antigene agent, molecular probes, and biosensors.^4^ Nielsen et al in 1991 designed a polyamide nucleic acid, by replacing the DNA backbone with an achiral polyamide backbone and found that in double‐stranded DNA these oligomers recognize their complementary target by strand displacement and the reason behind this displacement was due to the extremely high stability of the PNA‐DNA hybrids.^5^ Though they still have stability complications^6^ that can be improved by blocking or acetylating the N terminus, and apart from this, they are highly sequence‐dependent.

PNA is chemically stable than its biological counterpart's DNA and RNA, as they are polyamide‐based synthetic molecules, which are highly stable in an acidic environment, and moderately stable in basic conditions as well at high temperatures. They are also biochemically stable as they do not act as a substrate for *peptidases*, *proteases*, and *nucleases*, but on the contrary, their native nucleic acids are highly sensitive to these enzymes; due to PNAs well‐known biological and chemical stability. It facilitates synthesis, storage, purification, and applications. They also show excellent stability over a wide range of pH, but DNA and RNA are sensitive to these changes, and it also discriminates DNA or RNA in a single‐point mismatch. Thus, it acts as a more specific sequence binder. DNA/RNA is negatively charged, on the other hand, PNA lacks charges, and as a result of this during hybridization of PNA with DNA or RNA strands, PNA lacks electrostatic repulsion, which offers PNA a greater affinity towards its target. Introducing positive charge in PNA could be more suitable for the formation and stability of triplex and duplex helices. At higher salt concentration DNA‐DNA duplexes are generally stable while PNA‐DNA duplexes are highly stable in this condition. Hence, PNA is better alternative for their native biological counterparts for wide range of applications.^7^, ^8^

PNA is composed of adenine, uracil, guanine, and cytosine‐*N*‐acetic acids which offers them to be a capable precursor to RNA, as there are a lot of problems that exist with present thinking of RNA as genetic material; as RNA is known to be the primary genetic molecule prior to DNA‐based organisms on earth over 3.5 billion years ago. But following the researches and ease in the synthesis of PNA components and possibility in the polymerization of AEG (*N*‐[2‐aminoethyl] glycine) supports the hypothesis that PNA may be the first genetic material and may have been used before RNA‐based organisms arose, to transmit genetic information in primitive life.^9^ Thus, the study conducted by Banack et al^10^ further confirmed that cyanobacteria produces PNA backbone, and they detected AEG in axenic strains as well as environmental samples of cyanobacteria. Hence, the production by diverse taxa of cyanobacteria of AEG suggests that it may be a primitive feature that resulted early in the development of life on earth.

PNA can recognize specific sequences of DNA and RNA by obeying the Watson‐Crick hydrogen bonding arrangement, and these hybrid complexes exhibit unique ionic strength and unexpected thermal stability.^2^ These synthetic analogs display a wide range of applications in the biomedical domain. Along with a variety of applications, they also have few shortcomings such as issues with cellular uptake which can be dodged by modification of PNA or by making conjugates of PNA with cell‐penetrating peptides (CPPs). Thus, these modifications help them to improve their delivery inside the cells and offer them an application for targeted drug delivery.^11^ Nucleic acid biosensors have proved applications in genetics and biomedicine; PNA specifically can be used in the development of high‐performance affinity biosensors for applicability in DNA genotyping, though little has been studied about sensitivity and specificity.^12^ PNA was invented 28 years ago and has expanded briskly as helpful molecules in antisense technology and DNA hybridization. It is also used in diagnostics (in‐situ hybridization and polymerase chain reaction [PCR]‐based systems), and accompanies unique properties that have enlightened synthetic organic chemists to design and prepare other derivatives of these analogs with more refined properties.^13^

**FIGURE 2:**
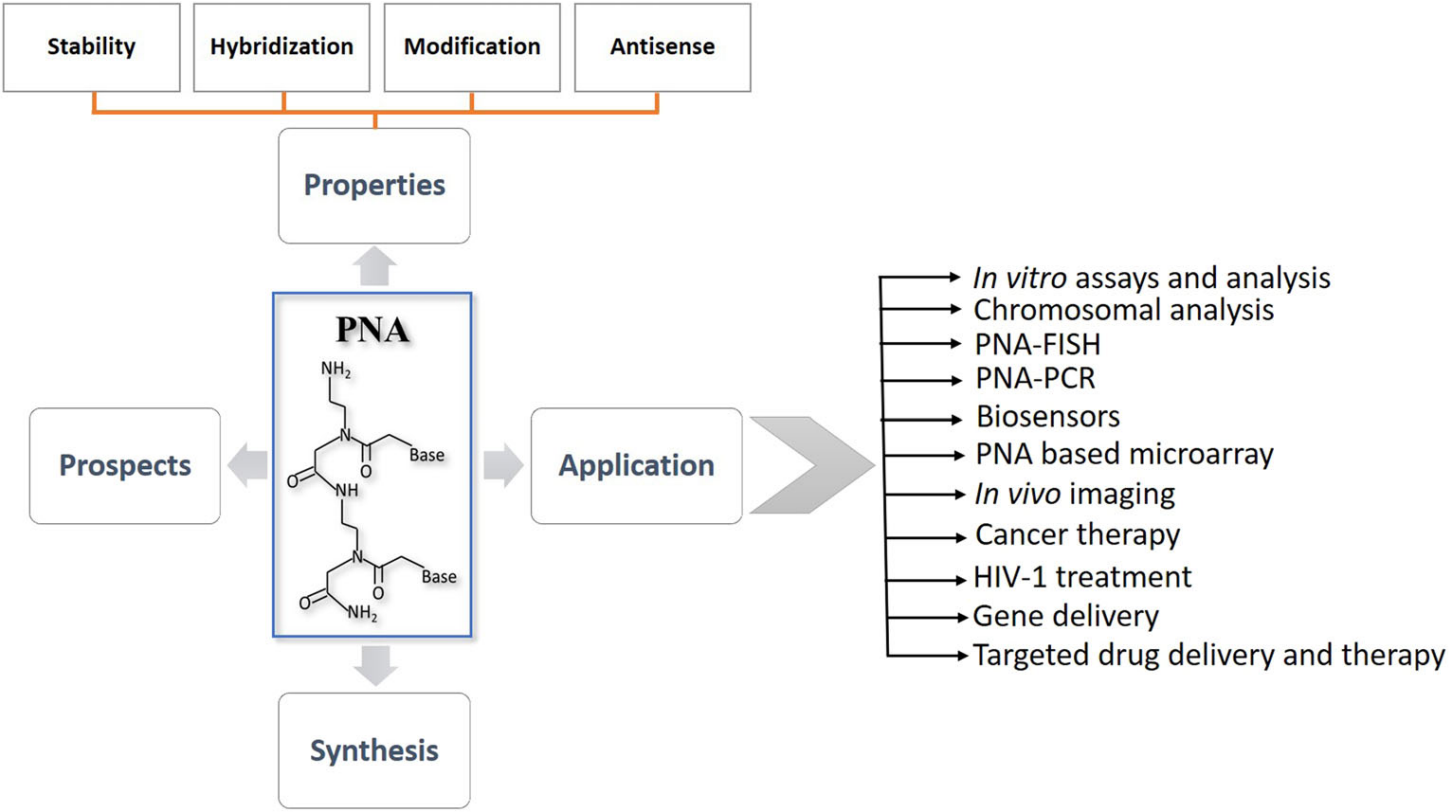
Diagrammatic illustration of details emphasized in this review

PNAs have the potential to be used as novel antibiotics, gene‐activating agents, and also as molecular probes in techniques like fluorescent in‐situ hybridization (FISH) for imaging and in biosensors for diagnostic purposes.^14^ PNA shows promising applications in detection techniques; wherein hybridization technologies based on FISH and novel approaches like matrix‐assisted laser desorption/lonization‐time of flight (MALDI‐TOF) utilizing PNA have gained lot of attention. PNA was a breakthrough among several efforts made to date for the development of gene therapeutic. Both in eukaryotes and prokaryotes, antisense activity of PNA as peptide conjugate has been reported with a positive result in the nerve cell‐line and in rat brain.^15^ Furthermore, the significance of single‐cell biology in basic research and translational medicine is growing at a faster pace; in which efficacious detection and isolation of specific subclasses using intracellular markers (IcM) are key for understanding their functional heterogeneity. The use of peptide nucleic acid probes (PNPs) as an IcMs against certain microRNA for cancer surgical margin predictions are gaining attention.^16^ Though nucleic acid analogs containing natural nucleobases on a modified backbone have been synthesized such as xeno‐nucleic acids, threose nucleic acids, locked nucleic acids (LNA), morpholinos, and so on, along with PNA,^17^, ^18^, ^19^, ^20^, ^21^ among all these designed oligonucleotides, PNA is a remarkable nucleic acid mimic as it is neither a nucleic acids nor a peptide.^22^ Furthermore, several reviews exist discussing PNA properties,^23^ chemistry,^24^, ^25^ and its modifications^26^, ^27^, ^28^ with details related to diagnostics and therapeutics, our review (Figure 2) specifically focuses on PNAs biomedical applications along with their properties, synthesis, and prospects.

## 2 PROPERTIES

Properties of PNAs offer vast applications in the field of biomedical research, including diagnosis using in vivo imaging and biosensors; in therapeutics used for antisense and antigene therapies^11^ which are elaborated in this section. Remarkable properties which are elaborated in this section.

### 2.1 Stability of PNAs

The complex structure of PNAs makes them more resistant to enzymes/chemicals such as *DNases* and *proteinases*, hence they do not degrade inside the cell and have high biostability which makes them a good candidate for in‐vivo study. Although its transportation within the cell is difficult due to its limited lipid membrane diffusion.^2^, ^11^, ^17^, ^29^ An experiment by Eriksson et al^6^ suggests that PNA with thymine monomer at 11 pH with 37 days half‐life undergoes *N*‐acyl transfer rearrangement, and at pH 9 or above they exhibit sequential degradation and *N*‐acyl transfer reactions. They have a high binding affinity and are specific to DNA/RNA in targeting due to their complex structure and stability, this property provides PNA as a choice for gene therapy‐based applications. These modifications provide more stability, thus, chemical alterations in combination with nanotechnology can help PNAs to improve their intracellular delivery, which was previously a significant challenge.^30^

### 2.2 Hybridization of PNA

**FIGURE 3:**
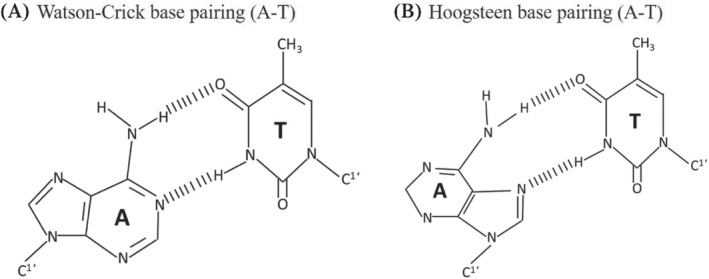
Difference between Watson‐crick and Hoogsteen base pairing in adenine–thymine (A‐T)

PNA hybridize at low ion concentration which exhibit remarkable stability towards complimentary oligonucleotides due to the fact that it does not show electrostatic repulsion and has flexible and neutral backbone.^11^, ^17^ PNA was designed to undergo Hoogsteen base pairing with dsDNA (double stranded DNA), and is proven to show effective DNA mimic with ability to partake duplex and triplex hybridization.^31^ Its hybrid complexes exhibit unique thermal stability and ionic strengths.^2^ PNA‐DNA duplex, properties are mostly studied in H‐TGTACGTCACAACTAA‐NH_2_ (a penta‐decamer).^32^, ^33^ PNA‐PNA, PNA‐DNA, and DNA‐DNA have melting temperature (*T*_m_) of 67°C, 51°C, and 33.5°C respectively. Among these duplex hybrids, PNA‐PNA is most stable.^34^ The DNA‐PNA or RNA‐DNA are more stable than the DNA‐DNA or RNA‐DNA duplexes due to the reason of high *T*_m_ values; typically, *T*_m_ value of 15‐mer PNA‐DNA duplex is approx. 70°C, and on the other hand *T*_m_ of DNA‐DNA duplex is approx. 50°C at pH 7, and a general rule suggests that *T*_m_ value of PNA‐DNA duplex is 1°C higher per base pair when compared with the *T*_m_ values of DNA‐DNA duplex in 100 mmol dm^−3^ sodium chloride. Table 1 gives the comparison in a generalized manner about the *T*_m_ values of 15‐mer RNA and DNA based duplex formation of PNA.^2^ (PNA)_2_‐DNA is formed by homopyrimidine PNA oligomers/PNA with high pyrimidine: purine ratio, via binding with complementary DNA. These triplexes are very stable complexes with *T*_m_ > 70°C (for decamer hybrid), binding of these complexes is governed by Hoogsteen and Watson‐crick base pairing (Figure 3).^5^, ^35^, ^36^, ^37^, ^38^ PNA‐DNA duplex for forming PNA‐DNA‐PNA/(PNA)_2_‐DNA triplex structure can be performed by strand invasion, in which one DNA strand is replaced by PNA of duplex complex but the replaced strand of DNA exists as D‐loop (single stranded).^39^

**TABLE 1:** General example of *T*_m_ values of 15‐mer duplex complexes formed by PNA, RNA, and DNA

Hybrids	Formation of duplexes (15‐mer)	*T*_m_
RNA	RNA‐PNA	72°C
	RNA–DNA	50°C
DNA	DNA‐PNA	69°C
	DNA–DNA	54°C

### 2.3 PNA modifications

Limited cellular uptake, poor membrane permeability, low aqueous solubility, and ambiguity in DNA binding orientation of PNA was a major challenge to use it as an antisense agent for in‐vivo*/vitro* studies.^40^ Hence, chemical alterations in PNA backbone improved the antisense properties along with many other properties of PNA for application in diagnostics, medicine, and molecular biology. PNA modifications by using different strategies such as introducing chirality into achiral PNA backbone, adding cationic functional group (to improve aqueous solubility), modifying linker/nucleobase (to control DNA/RNA binding at physiological conditions), and so on, are being considered. Furthermore, these modifications will result in better solubility in aqueous, enhanced cellular uptake, and many other properties; which will make it a better tool for biomedical applications.^11^ Apart from PNA backbone modifications, it can also be synthesized using artificial nucleobases, namely 2,6 diaminopurine, pseudoisocytosine, 2‐aminopurine, thiazole, hypoxanthine, thiouracil, and so on, which gives PNA unique feature such as increased affinity, selectivity for thymine, as a probe for detection of hybridization through fluorescence, and so on, these are just few examples with features of artificial nucleobases introduced in PNA.^41^, ^42^, ^43^, ^44^, ^45^ PNA backbone and nucleobase modification will result in enhanced properties of PNA which will provide new applications to the PNA with several features (Table 2). Hnedzko et al^52^ synthesized (M) modified PNA and found that, PNA are well‐suited ligands for dsRNA recognition in live cells and other biological systems. Hence, (M) modified PNA conjugates are promising probes, show excellent cellular uptake, and very less cytotoxicity; owing to wide range of biomedical applications of PNA.

**TABLE 2:** Modification of PNA for enhancing their properties. (A) Backbone modified PNA, (B) nucleobase modified PNA

S. No.	General name	Structure	Enhanced properties	Reference
*(A) Backbone modified PNA*				
1	Phosphono PNA	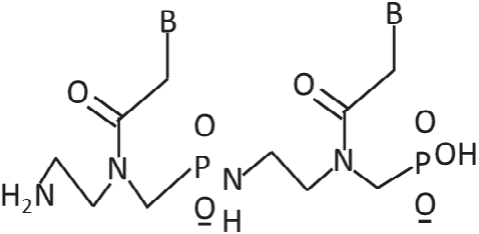	Antisense activity.	46
2	α‐Guanylated PNA	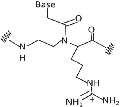	Cellular uptake properties while maintaining Watson‐Crick recognition with complementary DNA strand.	47, 48
3	α‐Amino methylene PNA	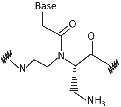	Cellular uptake.	49, 50
4	ϒ‐Amino methylene PNA	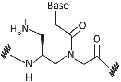	PNA‐DNA binding.	49, 50
5	Diethylene glycol PNA	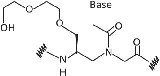	Water solubility and improved hybridization properties.	51
*(B) Nucleobase modified PNA*				
1	2,6‐Diaminopurine	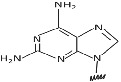	Affinity and selectivity for thymine.	41
2	Pseudoisocytosin	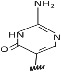	Mimics the C^+^ recognition pattern for triplex formation irrespective of surrounding pH.	41
3	2‐Aminopurine	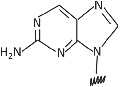	Hydrogen bond with uracil and thymine in the reverse Watson‐Crick mode and being inherently fluorescent, which can be used to study the kinetics of the hybridization process with complementary nucleic acids.	42
4	Thiazole	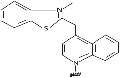	Forms PNA probe that fluoresced upon hybridization.	43, 44
5	Hypoxanthine	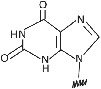	Form Watson‐Crick base pairs with A, C, T, and U and achieve multimutant specificity.	45
6	Thiouracil	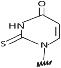	Invades dsDNA in antigene applications.	41

### 2.4 PNA antisense properties

PNA exhibit several properties desirable to be a good antisense agent.^40^ They identify DNA duplex homopurine sequences to which it binds by strand invasion, forming a very stable (PNA)_2_‐DNA triplex complex. In vitro studies indicate that PNA inhibits transcription and translation, and undergo complementary binding to RNA and DNA with high affinity and specificity; hence, these properties make PNA a “third generation” antisense and antigene agent. Owing to its superior properties, it can become a probe by replacing DNA for numerous investigation purposes.^2^, ^3^, ^53^ Among several delivery strategies explored till date, to improve the antisense properties of PNA, nanotechnology‐based strategies hold enormous potential. From prior studies, advances in nanotechnology by using nanoparticle‐based on polymer, carbon, and so on, for the delivery of PNA will improve the therapeutic applications of PNA. Hence, nanoparticle delivery of PNA can be implied in gene targeting (antisense) based strategies.^30^ The synthesized PNA exhibits enormous properties which make them a good antisense agent but they can be modified to enhance its antisense properties; one such example is Phosphono‐PNA (backbone modification), the added monomer gives the PNA improved antisense activity.^46^

## 3 SYNTHESIS OF PNA

**FIGURE 4:**
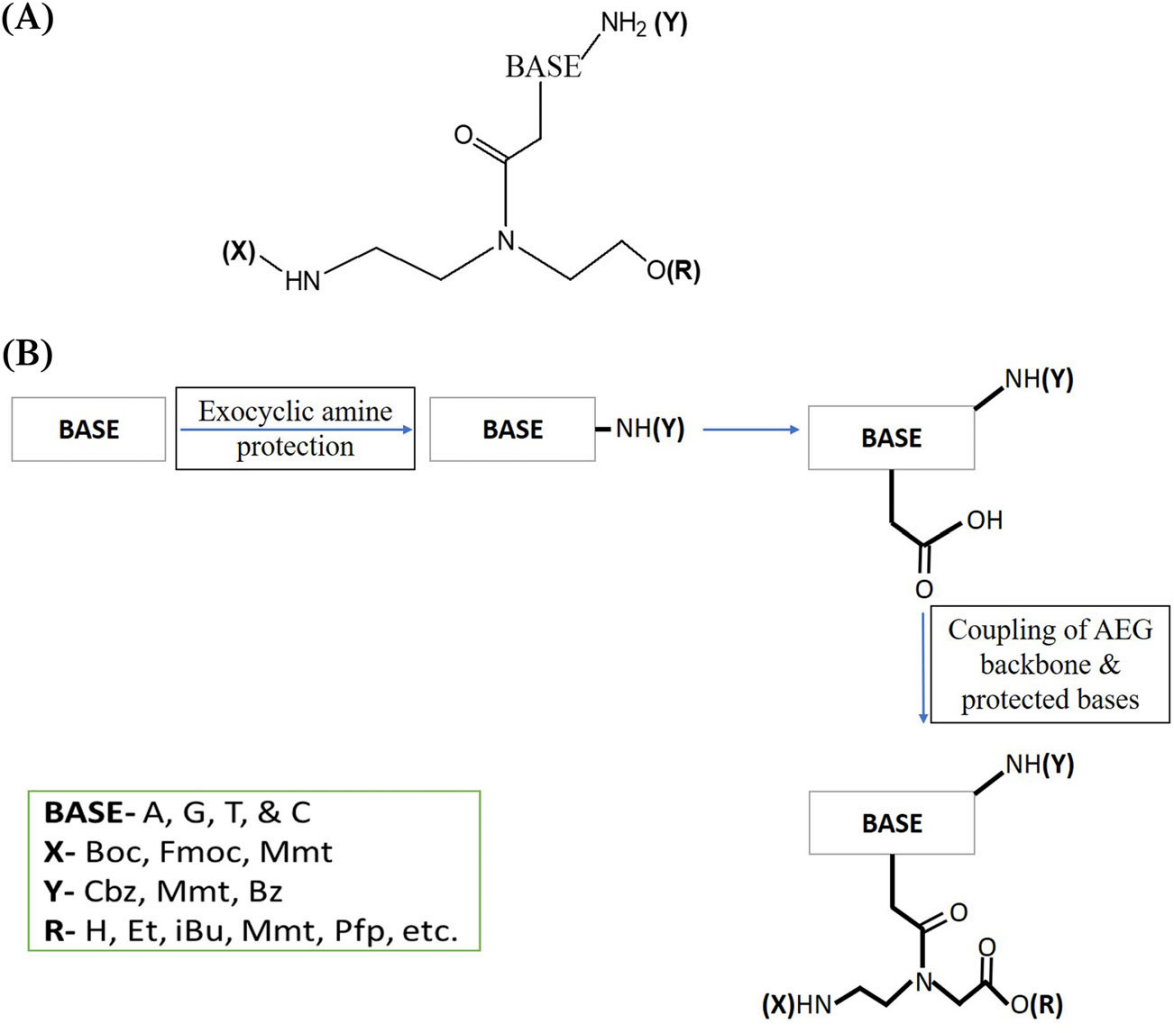
(A) General PNA monomer schematic illustration; (B) diagrammatic illustration explaining the synthesis procedure of PNA monomer

The pivotal requirement in PNA‐based research is its synthesis to form an oligomer.^7^, ^54^ PNA can be synthesized by self‐assembly and self‐organization process, and these processes are very versatile and easy, which produces a hybrid complex and also exhibits unique physicochemical and specific recognition properties. PNA monomer contains N‐protected AEG (Y), to which (X) protected nucleobase is attached (Figure 4A). Y and X are protecting groups in PNA and should be orthogonal that is X should be stable when Y is removed.^55^ PNA monomers are synthesized can be performed by convergent method.^56^ In this a suitable protected base and N‐protected AEG is initially synthesized, then their backbone is coupled with PNA nucleobase to get PNA monomer of interest (Figure 4B).

**FIGURE 5:**
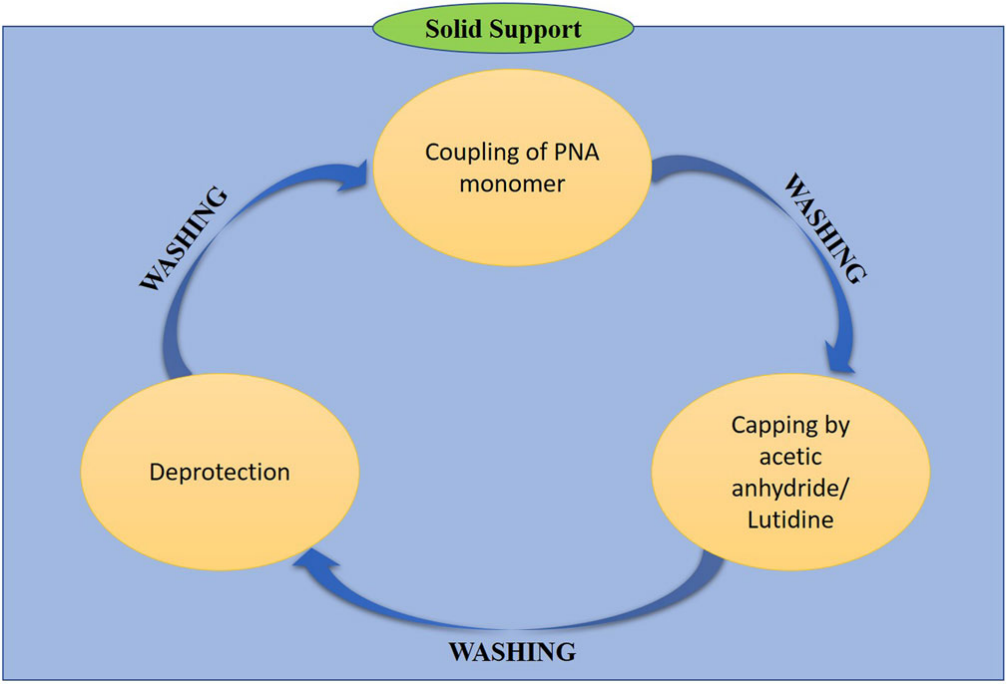
Diagrammatic illustration of basic solid‐phase PNA oligomer synthesis protocol

The protecting groups are always used in combination for the synthesis of PNA, to name a few group combinations are Boc‐Cbz, Fmoc‐Bhoc, Mmt‐Acyl, and Fmoc‐Mmt. Boc‐Cbz and Fmoc‐Bhoc PNA monomers are commercially available by Applied Biosystems (Foster City, United States) and can be used for PNA oligomer synthesis. Panagene^ⓒ^ is an international supplier of PNA since 2006, and the protocol utilizes a solid support via automated synthesis of PNA oligomer. Synthesis of PNA is a cyclic process and depends totally on the application for which the PNA is required. The synthesis of PNA oligomer starts by coupling of PNA monomers, then capping by acetic anhydride/lutidine, by concluding with deprotection (Figure 5). On the contrary, synthesis of Fmoc PNA monomer though was not cost‐effective, but face difficulties in mass production, needs a coupling reagent, and has purification complications leading to low yield. The solution to this was brought forward by Panagene^ⓒ^ by synthesizing Bts monomer, as the synthesis of this was cost‐effective, and does not require coupling reagent, has ease in mass production, and easy purification process leading to high yield.^57^

## 4 APPLICATIONS OF PNA

As PNA has better chemical, physical, and biological properties relative to nucleic acids, they find applications in various fields while showing promising development of diagnostic devices, therapeutic agent, molecular tool for nucleic acid manipulations, and as tool in biomedical applications since its discovery in 1991.^2^, ^3^, ^58^ PNA is synthesized both at nano‐ and micro‐scale levels and have potential applications in various fields for making nanostructured devices for biochips, sensors, and microarrays.^59^ Beavers et al^60^ in their study reported the in‐situ synthesis of PNA from porous silicon (PSi) and confirmed the establishment of a novel PNA‐PSi platform with broad utility in drug delivery and biosensing. Ma et al^61^ have explored CPPs‐PNAs by antisense inhibition of intracellular bacteria, and their results suggested that electroporation is the best approach for CPP‐based delivery. PNA exhibits potential antisense applications in biomedical field as they hybridize with neutral nucleic acids to form complexes and show enhanced biochemical properties. Thus, they have emerged as a tool for cancer diagnosis and therapy by utilizing a combination of nanobiotechnology with PNA biotechnology. PNAs have also proved to have potential applications in the cytogenetic and genetic diagnostic procedures.^22^ Furthermore, PNA‐based nanoparticles can also be used to improve the efficiency of drug delivery in anticancer therapy.^8^

### 4.1 PNA applications in diagnosis

#### 4.1.1 In‐vitro assays and analysis

PNAs unique physicochemical properties have led to the development of in‐vitro diagnostic assays for advance routine clinical tests and environmental monitoring utilizing PNA technology. Apart from these applications PNAs also has an impact on other fields of cytogenetics and industrial microbiology.^62^ Su et al^63^ have developed an enzyme‐based colorimetric assay for nucleic acids utilizing PNA as a probe and have validated the assay on different DNA as well as on total RNA samples extracted from two human cancer cell lines of lung and HeLa cell line for detecting miRNA. This study signifies PNA assays have better detection and sensitivity, introduces additional robustness, and avoids labeling of sample, making them better assays over other colorimetric analyses. Furthermore, there are many more in‐vitro diagnosis techniques that utilize PNA, namely chromosomal analysis, PCR, FISH, microarray, and biosensors; that are elaborated in the below sections.

#### 4.1.2 Chromosomal analyses

PNA is an excellent probe for molecular hybridization because of its unique hybridization properties. PNA‐based technology is developing and can replace FISH for human chromosomes detection in different tissues using in‐situ detection, which are reported by many researchers. PNA‐based hybridization technology utilizes PNA as a probe, having greater potential for chromosomal investigations.^29^, ^64^, ^65^ Chen et al^66^ synthesized 42 PNA probes for chromosome‐specific probe identification, which resulted in 10 PNAs were specific to human chromosomes 1, 2, 7, 9, 11, 17, 18, X, and Y. Their results also suggested that probes of PNA are very much capable of detecting trisomies in humans, and also are used for chromosome “bar‐coding” as sequence‐specific stains. FISH based on PNA is widely being investigated and has shown promising application for chromosomal analysis, as traditional techniques require denaturation of DNA by heat in formamide along with moderate temperature for longer hours to hybridize probe to its specific target.^67^

#### 4.1.3 PNA in PCR

In biomedical research PCR is a widely used technique for DNA amplification of targeted sequence, it generates thousands of copies for enormous applications. The methods based on PNA are used to modulate PCR, detecting genomic mutation/capturing nucleic acids.^22^ Yu et al^68^ designed a “modified PNA‐PCR method” to screen KRAS mutation in cancer patients, this mutation shows resistance to epidermal growth factor receptor (EGFR) target therapy. From the results it was evident that this modification in PCR using PNA was an accurate and sensitive method to screen pancreatic cancer patients with KRAS mutation, apart from screening, this method also has potential to quantify KRAS mutant DNA to envisage response of cancer patients towards therapy and monitoring of disease progression. PNA‐based PCR clamping is used for minor allele identification in blood chimerism, and on the other hand, normal PCR is not capable of identifying it.^69^

**FIGURE 6:**
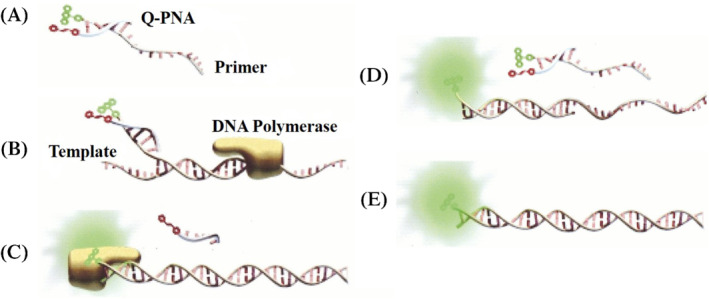
Illustration of basic Q‐PNA‐based PCR principle: (A) Q‐PNA labeled with quencher hybridized at 5′ end of the forward primer labeled with Fluor which will quench fluorescence. *T*_m_ value of Q‐PNA‐primer duplex complex is ranged between temperature of PCR annealing and extension stages. (B) In initial stage of thermo‐cyclic reaction, denature DNA is hybridized by 3′ end of forward primer and DNA polymerase elongates the primer. (C) Reverse primer which is not illustrated in the figure, will displace the Q‐PNA and incorporate 13‐base‐Q‐PNA binding region into amplicon by initiating transcription of the reverse strand. (D) Later in annealing stage, forward primer fully anneals to reverse strand and report its presence in real‐time analysis because excess primer is quenched by the Q‐PNA. (E) This is end product: a double stranded piece of DNA labeled fluorescently at its one strand. *Source*: Reproduced from Fiandaca et al, Genome Research, 2001^71^

PNA‐based real‐time PCR kits are highly sensitive and specific with very excellent detection limit, having no cross reactivity, stable reproducibility, and self‐life of more than 8 months, for the detection of carbapenemase genes.^70^ Furthermore, PNA‐based quantitative PCR (Figure 6) are also used for rapid and sensitive detection of multiple sclerosis‐associated retrovirus, which is responsible for neuro‐inflammatory complications such as multiple sclerosis.^72^ Nowadays, PNA‐based probes in PCR methods for highly sensitive detection of genetic drivers such as KRAS are in high demand, as normal quantitative PCR are not very sensitive for genetic mutation determination.^73^ Clamping of PNA in PCR is used for diagnosis of McCune‐Albright syndrome which affects skin, endocrine tissues, and bones of effected person.^74^ PNA clamping is ultrasensitive for detecting EGFR gene mutation when compared to traditional way of direct sequencing in non‐small cell lung cancer patients.^75^ In addition to this, PNA‐LNA conjugated PCR clamping assay are being used to detect EGFR gene mutation from pleural fluid sample of non‐small cell lung cancer patients, and the results obtained suggest that these conjugate based PCR is highly sensitive than pre‐existing PCR techniques which are based on direct sequencing.^76^, ^77^

#### 4.1.4 PNA‐FISH

Fluorescent PNAs are an important tool in the biomedical domain for utilization in the FISH technique as a probe. Hnedzko et al^78^ presented a simple protocol for labeling N‐terminus of PNA with HiLyte Fluor 488 in the last stage of PNA synthesis, which resulted significantly brighter and photostable in microscopy experiment when compared with PNA‐carboxyfluorescein. Santos et al^79^ optimized the PNA probe (EUB338) targeting the bacteria by using flow cytometry for studying the three major factors responsible for hybridization that is time, temperature, and formamide concentration. Hence, their results signify that universal PNA‐FISH procedure does not apply to different microorganism's families as they require different hybridization parameters. In 2019 Huang et al^80^ reported PNA‐FISH‐AFC (PNA‐FISH‐Acoustic Flow Cytometry) for bacterial detection in blood culture; as it was important to develop a rapid technique to diagnose bacteremia, which is a bacterial infection in the bloodstream caused by bacteria such as *Escherichia coli*, *Klebsiella pneumoniae*, or *Pseudomonas aeruginosa*, and so on, and conventional techniques like PCR‐based film array assay and MALDI‐TOF take 7‐24 hours for complete diagnosis. But PNA‐FISH‐AFC method was found to be more appropriate and had potential time advantages. PNAs are hydrophobic and can also penetrate the mycobacterial cell wall; considering this point, Kim et al^81^ developed a FISH‐PNA probe method to differentially label *Mycobacterium tuberculosis* and non‐tuberculosis mycobacteria for detection in clinical respiratory specimens. The results obtained from their study suggest that dual‐color FISH with PNA probe was highly specific, but less sensitivity for detection and identification of mycobacterium. Hence, due to this reason, this method is best suited for culture confirmation.

Q fever endocarditis and vascular infections are very common infections caused by bacteria *Coxiella burnetii*, and FISH is used for diagnosis of this. Prudent et al^82^ compared the conventional FISH method with PNA‐FISH for diagnosis of Q fever endocarditis and vascular infections in real‐samples collected from patients. Their result suggests that the FISH was approx. 43% sensitive and PNA‐FISH was approx. 60% sensitive. Hence, it suggests that PNA‐FISH is more sensitive than the FISH method for detecting *C. burnetii*. Naturally, biofilm is composed of a variety of species,^83^ these multispecies biofilms can be held responsible for complications in different areas, namely drinking water distribution system biofouling, food processing environment contamination, biomedical complications, and infections from the device in humans.^84^, ^85^, ^86^ Almeida et al quantified and visualize multispecies biofilm populations by utilizing PNA‐FISH and PNA‐FISH‐confocal laser scanning microscopy (CLSM) as a tool. The result suggested that PNA‐FISH was very efficient in quantification and microbial distribution of multiple species, and when this was combined with CLSM, it was more effective then PNA‐FISH.^87^

#### 4.1.5 PNA‐microarray

**FIGURE 7:**
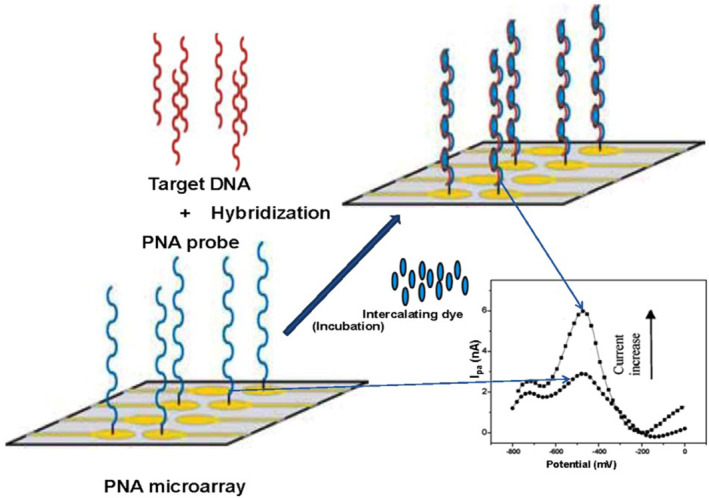
Diagrammatic representation of PNA‐based microarray basic set‐up for fabricating gold electrode based chip surface, to detect the hybridization events. *Source*: Reprinted by permission from Elsevier: Elsevier, Bioelectrochemistry, Application of peptide nucleic acid towards development of nanobiosensor arrays, Singh, R. P., Oh, B.‐K., & Choi, J.‐W. Bioelectrochemistry. 2010^90^

PNA microarray is more preferred over DNA chip in gene analysis, but a chemical synthesis of PNA microarray is a laborious process and very sophisticated art. Due to this reason, Wu et al^88^ developed a fully automated synthesizer to fabricate the PNA microarray through photolithography. Yang et al^89^ developed a PNA microarray that has improved performance when compared to DNA microarray. In their study, they have synthesized PNA and used it as building blocks for developing high‐density PNA microarrays and result suggest that the PNA microarray detected single and multiple base‐mismatches with high efficacy. DNA‐based microarray is very effective with a very few fallbacks, such as selectivity, sensitivity, and stability in varying conditions; the solution to this problem is PNA‐based probe due to its enormous properties. PNA‐based chips are very rapid, highly reproducible, accurate, reusable, and have long storage capacity. Figure 7, represents basic PNA‐based microarray, which illustrates PNA immobilization on the gold electrode chip surface. Then is hybridized with target DNA, and after target DNA gets hybridized, intercalator “Hoechst 33258” is introduced and incubated for binding with the PNA‐DNA complex. After incubation, the hybridization event is evaluated using the electrochemical analysis method.^91^, ^92^

**FIGURE 8:**
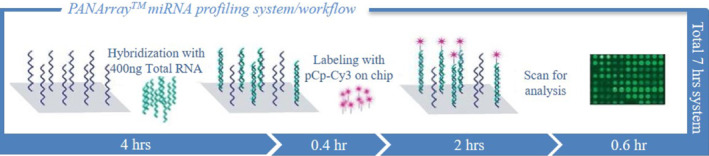
PANArray™ miRNA microarray system: In this system glass slide arrayed with miRNA specific PNA probes were hybridized with total RNA. Labeling with pCp‐Cy3 at 3′ end by enzyme ligation, to form a chip for analysis. Processing and analysis using standard software and scanning equipment. This system takes only 7 hours for the analytical process (form fabrication to analysis) to give an excellent result. *Source*: Reprinted by permission from Springer Nature: Springer Nature, BioChip Journal, A PNA microarray platform for miRNA expression profiling using on‐chip labeling technology, Kim, H., Choi, J., Cho, M., & Park, H. BioChip Journal. 2012^93^

Researchers have investigated a new rapid chip technology based on PNA‐microarray for cancer diagnosis, prognosis, and research. For this, they have hybridized unlabeled RNA on PNA microarray and further labeled this with pCp‐Cy3 by enzyme ligation on the chip. The designed chip was commercialized with trade name, PANArray™ miRNA (Figure 8), which demonstrated excellent reproducibility and low cross‐hybridization for let‐7 and the miR‐181 families of miRNA. Thus, the results of this study suggest that this microarray is rapid chip‐based technology for expression analysis with high fidelity.^93^ Microarray technologies are widely used for biomedical studies. Endoh et al^94^ prepared PNA‐based array and investigated it for capture ability of microRNAs, and showed that 10‐mer PNA probe microarray was highly sensitive than PNAs biological counterpart DNA as a probe for miRNAs targeting. For cancer diagnosis, miRNA plays a crucial role as a promising biomarker; low levels of miRNA in blood hampers their use for cancer diagnosis, and to overcome this issue development of the ultrasensitive analytical method is the need of current scenario. For the same, Jolly et al investigated PNA as probe immobilized on the gold electrode to capture miRNA, by utilizing dual‐mode detection that is electrochemical impedance spectroscopy and thiolated ferrocene. Their study suggests that the dual detection approach for miRNA was highly sensitive and were able to distinguish between mismatched target sequence of miRNA, and can be further exploited for the development of microarray platforms.^95^

#### 4.1.6 Biosensors

Biosensors based on nucleic acid have increasing applications in genetics and biomedical; for example, DNA genotyping and cancer biomarker detection by identifying single base mutations in a target nucleic acid at an extreme‐low concentration by using PNA‐based biosensors, which have an excellent affinity, sensitivity, and specificity.^12^, ^96^ Singh et al^90^ reported applications of PNA for nano‐based biosensor (nanobiosensor) array, as PNA possess physicobiochemical properties which enables the establishment of new detection approach that is more reliable and faster than traditional biosensor arrays due to this reason PNA is used for the development of chips/sensors/arrays for the diagnosis and therapeutics. PNAs are used in sensing technology which has biomedical applications such as, diagnosis stratification of diseases, and prognosis, and is better as well as inexpensive than traditional technologies. Due to PNAs exceptional biophysical properties, fluorogenic‐PNA can be preferred over molecular beacons based on DNA for sensing in a cell‐free environment; hence, cellular uptake is not necessary.^97^ Hamidi‐Asl et al^98^ developed a genosensor (electrochemical detector) by labeling PNA with indigo carmine (IC) for detection of tumor suppressor gene p53 (15‐mer short sequence), using IC label in PNA for the first time and the result obtained from this study indicates that PNA sensors have a good logarithmic dependency.

Saadati et al^99^ reported the applications of PNA in biosensor as a bioreceptor; PNA is used as a diagnostic probe by fabricating it on the surface of electrochemical/optical biosensors, which has application in identification of various analytes of interest; further these biosensors can be employed for recognizing DNA, RNA, bacteria, and so on. Bazin et al^100^ reported in their review that PNA are attractive receptors, and recent developments in nanostructured and interface materials play a key role in future biosensor technology. Teengam et al^101^ developed a paper‐based electrochemical PNA biosensor. In this, the PNA was labeled with anthraquinone‐labeled 28 pyrrolidinyl peptide nucleic acid probe (AQ‐PNA) and further, the electrode was modified by graphene‐polyaniline (G‐PANI) for the detection of human papillomavirus (HPV) type 16 DNA. The detection was done on real DNA samples, after PCR amplified DNA samples from the SiHa cell line. The result suggests that this device is inexpensive, can be incinerated with ease, and is reliable for detection of the primary stage cervical cancer by measuring the amount of HPV type 16 DNA.

Lab‐on‐chip based technologies are very high performing and cost‐effective; they are playing key role for advancing futuristic point‐of‐care diagnostic devices. Diagnostic work based on printed circuit board (PCB) that can be called as Lab‐on‐PCB, have recently reemerged as a promising technology for mass‐manufacturing of integrated microsystem technology.^102^, ^103^ Till date for genetic analysis microsystems, such as DNA, RNA, miRNA, and cRNA; are the main objectives. Keeping the above viewpoint, Jolly et al developed lab‐on‐chip‐PCB biosensor based on PNA as a probe for genetic diagnosis by DNA quantification. The result showed higher selectivity and sensitivity than the pre‐existing techniques for the same purpose.^104^ Xuan et al^105^ developed an economical and portable DNA recognition device that is an ultrasensitive DNA electrochemical sensor, which does not require immobilization of probe. There are two advantages of this method one is diffusivity between ferrocene‐PNA and ferrocene‐PNA hybridized to DNA, and the other one is the DNA polymerase activity of strand‐displacement. Thus, the results obtained from this study suggested 2‐3 folds higher detection sensitivity than other pre‐existing DNA biosensors, which are not based on immobilization. Hence, extraordinary sensitivity and ease in operating offered this method with a promising substitute for the development of miniaturized, integrated, cost‐effective, and portable biosensor devices for DNA detection.

#### 4.1.7 In‐vivo imaging

Sonar et al^106^ developed a method to detect KRAS2 mRNA hybridization using PNA‐fluorescence in lung cancer cells. They labeled PNA with thiazole orange (TO) dye at 5′ end for making PNA‐IGF1, this labeling with dye enhanced the fluorescence quantum yield. Their study showed that PNA‐IGF1 with TO shows less fluorescence, but hybridization of PNA‐IGF1 with KRAS2 RNA forms KRAS2 PNA‐IGF1, which has raised fluorescence multiple folds. Shigeto et al^107^ designed a PNA‐DNA probe, in which PNA and DNA conjugated with fluorescein isothiocyanate for detection and Dabcyl as quencher probe respectively; for imaging analysis of EGFR mutation especially the following three EGFR mutations exon19del E746‐A750, T790M, and L858R in lung cancer cells. Chen et al^108^ designed an assay based on PNA, tagged with a fluorophore for detection of BRAF V600 mutations with excess wild type background in melanoma samples. The tagged PNA act as both PCR clamp and sensor probe; the result obtained from this study has shown better sensitivity than traditional PCR plus Sanger sequencing. Hwang et al^3^ developed a PNA probe labeled with fluorescent markers for convenient, rapid, and accurate detection of filaggrin (FLG) mutation. These mutations are of two types heterozygote and homozygote FLG mutations and are found in patients of atopic dermatitis (chronic inflammatory skin disease). Hence, these detection techniques will allow early prevention and will lower the incidence rate of atopic dermatitis.

### 4.2 Therapeutics utilizing PNA

PNA has a variety of properties such as antisense agent, antigene agent, versatility, affinity for targeting nucleic acids, and physiological stability. This offers PNA to be a potential therapeutic agent. Modifications in PNA have improved its potency for further developments in preclinical studies for drug delivery and has reached new statures.^109^, ^110^ It can be used as a research tool for gene regulation and targeting. The D‐loop formation during PNA hybridization to form a triplex complex has the ability to initiate transcription and induce gene expression. PNA‐based gene regulation will have important biomedical implications for the treatment of many deadly diseases such as cancer, genetic disorder, and age‐related diseases.^39^ PNA in general cannot cross the blood‐brain barrier as it is permeable to only lipophilic molecules of molecular weight < 600 Da. Hence, conjugates of streptavidin (SA) and OX26 monoclonal antibody with PNA represent optimal antisense molecules for drug delivery to the brain for the treatment of Alzheimer's disease and cerebral AIDS.^111^ Kaushik et al^112^ reported that anti‐TAR PNA conjugation with membrane‐permeating peptide inhibits transactivation of the HIV‐1 (Human Immunodeficiency Virus Type 1) LTR (long terminal repeat), which results in reduced HIV‐1 virions production in chronically H9 cells infected from HIV‐1. Chaubey et al^113^ designed PNA_TAR_‐transportan conjugate, which has speedy cellular uptake and exhibits potential virucidal and antiviral properties. Neamine part from the aminoglycoside antibiotic neomycin B can be conjugated to a 16 mer PNA targeting HIV‐1 TAR RNA. The TAR RNA is the binding site of the viral protein TAT. Attachment of the neamine core allows cellular uptake of the PNA and results in effective inhibition of HIV‐1 replication. Neamine is a polycationic moiety which provides greater solubility to PNA and also gives it a unique property to cleave RNA, the conjugate is specific to its target site and is only functional at physiological concentrations of Mg^2+^. Hence, this conjugate of PNA with neamine has a potential therapeutic application.^114^ Zeng et al^115^ designed Tat‐PNA‐DR to target the direct repeat (DR) sequence of Hepatitis B virus (HBV); this was validated in‐vitro using HepG2.2.15 cells, and in‐vivo in acute hepatitis B mouse model by targeting LTR‐DR of HBV‐RNA. Targeting using this conjugate inhibited HBV replication in the in‐vitro study, and it also displays low cytotoxicity, less immunogenicity, and elevated stability in serum; an 80% decline in HBV‐DNA was observed in the in‐vivo studies. Hence, from these results, they have concluded that PNA conjugated drugs can be a promising candidate for HBV treatment.

Browne et al^116^ reported for the first time, efficacy of PNA and their conjugates for the treatment of amyotrophic lateral sclerosis (ALS) by specific cellular pathologies, as ALS is a neurodegenerative disorder, which affects the spinal cord and nerve cells. Ricciardi et al^117^ reported the details of PNA as a site‐specific gene‐editing tool due to its hybridization properties they form a stable triplex complex, which has potential for genome‐specific modification and DNA repair. PNA‐based strategies for gene editing can positively correct the human diseases caused due to mutations in preclinical studies. Bahal et al^118^ reported γPNA‐mediated gene editing for correction of anemia in human β‐thalassemic mice model using nanoparticle‐based delivery system. This in‐vivo study result shows that the nanoparticle delivery system has low off‐target effects and stem cell factor, γPNAs and nano‐system based delivery will offer new treatment strategies for the genetic disorder related to the blood which can be treated safely by intravenous administration. Dorn et al^119^ designed phosphonic ester PNA by modifying the side chain of PNA to interfere with gene function and to modulate cellular pathways according to the demand for therapy, as AEG PNA hampers in‐vivo applications. Their result suggests that PNA is a powerful antisense molecule which when directed against medaka (*Oryzias latipes*) *six3* gene in fish embryos it was able to knock down the desired gene, which resulted in exact *six3* phenotype.

## 5 FUTURE PROSPECTS AND CONCLUSIONS

PNA is a synthetic RNA/DNA analog with mimicking attributes of DNA; they exhibit unique features, such as higher specificity and affinity, poor solubility, can form hybridized complexes with high stability; its structure can be modified by targeting strand invasion and self‐tendency for aggregation. These distinctive features of PNA have given it a remarkable advantage over its corresponding analogs, to extend state‐of‐the‐art development of diagnostic and therapeutic tools in the biomedical domain after its synthesis in 1991. PNA is highly used for diagnostic applications such as in vivo and in vitro analysis. PNA biosensors have evolved recently and have shown improved detection of analytes when compared to conventional biosensors. Hence, we suggest researchers to utilize nanotechnology for enhancing clinical diagnosis attributes of PNA fabricated biosensors for detecting human diseases, which will open new avenues for future research in the making of cliniconanobiosensor and nanobiosensor arrays for clinical diagnosis. In vitro assays based on PNA are widely used for clinical diagnosis, but they have less detection sensitivity at nanomolar concentration and need improvement.^63^ For this, we recommend that it can be achieved by employing nanobiotechnological applications. PNA‐based diagnosis has achieved great heights and apart from biomedical applications, they are also used for the detection of small‐size toxins in the environmental monitoring and food analysis industries.

PNA is a synthetic molecule that shows highly specific binding to target gene sequence due to which they are an appropriate candidate of interest in biomedical and biotechnological context.^3^ Tagalakis et al^120^ developed a nanovesicle (∼90 to 140 nm) complex made from giant unilamellar vesicle to efficiently deliver the drug to the target site of gene therapy by therapeutic nucleic acids, as targeted delivery is one of the challenging aspects in PNA‐based gene therapy. PNA in the therapeutic application was a challenge because of its intercellular delivery which is now improved utilizing nanoparticle‐based strategies for efficiently delivering the PNA conjugated drugs, to the cellular environment.^121^ Furthermore, to extend the oligomeric family, many researchers are working to develop a new synthetic analog with properties more sophisticated than PNA, such an example is the development of AApeptides (a new class of peptidomimetics) which is based on chiral PNA backbone.^122^ This class of peptide shows application in material science and biomedical domains because of their unique and few properties similar to PNA. A large number of transfection strategies for PNA to overcome intercellular uptake have been developed utilizing microinjection, electroporation, conjugation with lipophilic moieties, and so on, to date and is still developing at a faster pace.^53^

The current situation of global viral pandemic (COVID‐19) is threatening the society; PNA and nanoparticle functionalized PNA can be used for the manufacturing of antiviral PPE (personal protective equipment) kits due to this reason PNA shows great potential as an antiviral agent.^123^ PNA is not present in the human body, and the clearing mechanism of this from the human body is not known, and this may lead to systemic toxicity. Thus, there is an urgent need to lay attention towards the toxicological aspect of PNA and their conjugates. The search of synthetic nucleic acid with better properties such as higher biological stability, enhanced cellular uptake, and strong binding affinity with complementary DNA/RNA is still going on, but to date, PNA is the best synthetic nucleic acid analogs and can trigger the ideas for next‐generation novel technologies.^58^ Hence, from a detailed study of PNA, we can suggest that researchers should lay their focus on engineering the PNA by functionalizing them with different nanoparticles (carbon‐based, quantum dots, metal and metal oxides, liposomal, and dendrimer nanoparticles) for improved and high‐end applications. Due to the higher prevalence of lifestyle diseases in the society^124^ and PNA has well‐developed applications towards diagnostics and therapeutics. Hence, our review emphasizes research professional's attention towards the biomedical domain, for the treatment and diagnosis of deadliest lifestyle diseases, like cerebrocardiovascular diseases, cancer, diabetes, and so on, by utilizing PNA. Furthermore, PNAs plausible application potentialities towards the agriculture and environmental domain may be examined which are scantly reported or are in the infancy. To conclude our review gives a comprehensive picture on detailed biomedical application of PNA, along with their properties, synthesis, and prospects.

## PEER REVIEW INFORMATION

*Engineering Reports* thanks Nuno Filipe Azevedo and other anonymous reviewers for their contribution to the peer review of this work.

## AUTHOR CONTRIBUTIONS

Kshitij RB Singh contributed to the conceptualization, data curation, formal analysis, investigation, methodology, project administration, resources, validation, visualization, writing the original draft, review, and editing. Parikipandla Sridevi contributed to the conceptualization, resources, supervision, writing the review and editing. Ravindra Pratap Singh contributed to the conceptualization, project administration, resources, supervision, writing the original draft, review, and editing.

## CONFLICT OF INTERESTS

The authors declare no potential conflict of interest.
